# A phylogenetic classification of the Je language family

**DOI:** 10.12688/openreseurope.19346.1

**Published:** 2025-01-30

**Authors:** Fabrício Ferraz Gerardi, Tim Wientzek, Jonas Gregorio de Souza, Ivan Roksandic, Fernando Orphão de Carvalho

**Affiliations:** 1 University of Tübingen Department of Linguistics, Tübingen, Baden-Württemberg, 72074, Germany; 2 Pompeu Fabra University Department of Humanities, Barcelona, Catalonia, Spain; 3 The University of Winnipeg Department of Anthropology, Winnipeg, Manitoba, Canada; 4 Federal University of Rio de Janeiro National Museum, Rio de Janeiro, State of Rio de Janeiro, Brazil

**Keywords:** phylogenetics, historical linguistics, macro-je

## Abstract

**Introduction:**

This study investigates the Je language family, addressing a significant gap in previous research by applying quantitative methods to its classification.

**Dataset:**

The dataset comprises 516 concepts from 14 languages, primarily sourced from Swadesh lists and culturally relevant terms, providing a robust foundation for phylogenetic analysis.

**Methods:**

Bayesian phylogenetic inference and NeighborNet methods were employed to analyze the dataset. These approaches enabled the reconstruction of evolutionary relationships within the Je family, facilitating the identification of language divergence patterns and their historical dynamics.

**Results:**

The analysis reveals well-supported Northern, Central, and Southern subgroups within the Je family, demonstrating clear geographical clustering. The phylogenetic tree aligns with existing hypotheses while offering new insights into the family’s structure.

**Discussion:**

The findings were contextualized within pre-Columbian archaeological frameworks, drawing parallels between linguistic divergence and material culture. These connections support the hypothesis that the Macro-Je language family’s development aligns with distinct cultural and geographical distributions observed in archaeological records.

**Conclusion and Future Directions:**

This study affirms the genetic coherence of the Je family and highlights opportunities for future research, including the incorporation of non-Je languages and expanded datasets to refine the understanding of this diverse linguistic group.

## Plain Language Summary

This study examines the Je language family using quantitative methods to improve its genetic classification. Researchers analyzed 516 words from 14 languages, mostly from Swadesh lists and culturally important terms, to trace their historical relationships. By applying Bayesian phylogenetic inference and NeighborNet methods, they identified patterns of language divergence and subgroup connections.

The findings align linguistic changes with archaeological evidence, suggesting that the evolution of the Je family reflects historical cultural and geographic patterns. The study confirms the existence of Northern, Central, and Southern subgroups within the Je family, reinforcing previous research while offering new insights. The results also support the genetic unity of the Je family and highlight future research directions, such as including more languages and expanding the dataset for a deeper understanding.

## 1 Introduction

This paper represents a first effort to apply phylogenetic classification to the Je language family, addressing a significant gap in Macro-Je studies, as quantitative approaches have been notably absent in previous research. Phylogenetic methods, which are well-established in linguistics, have been widely used to elucidate the classification and dating of various language families, such as Indo-European ( Chang *et al.*, 2015), Dravidian ( Kolipakam *et al.*, 2018), Alor Pantar ( Kaiping & Klamer, 2019), Mixtecan ( Auderset *et al.*, 2023) and Tupi-Guarani ( Ferraz Gerardi *et al.*, 2023). These methods are particularly suited for testing hypotheses related to prehistory, linguistic evolution, and evolutionary dynamics ( Greenhill *et al.*, 2020).

After Von den Steinen (1886), von Martius (1867) introduced the term “Je” to designate this language family based on lexical and cultural comparison (see also Ehrenreich, 1891). He derived the name “Je” from a common ending found in many of the group’s names.

The Je language family today comprises about fifteen languages ( Hammarström *et al*., 2024), some with multiple dialects (see, e.g., Gildea & de Castro Alves, 2020: 50), all spoken in Brazil and geographically distributed broadly. Many Je languages have become extinct, and, for most, only limited data is available ( Gildea & de Castro Alves, 2010; Nikulin, 2020; Nimuendajú, 2017). Davis (1966), based on phonemic correspondences, proposed internal relations among Apinajé, Timbira, Xavante, Kaingang, and Suyá. This was an important step, as it demonstrated the existence of geographical clusters within the Je family: Northern (Timbira, Apinajé, and Suyá), Central (Xavante), and Meridional (Kaingang).

The proposal for the Macro-Je phylum ( Mason, 1950; Schmidt, 1926), consisting of the Je family and several smaller families, was supported by studies such as Rivet (1924) (see also Loukotka, 1932; Loukotka, 1937; Loukotka, 1939; Loukotka 1942[1944]), which provided further evidence for this phylum, including languages such as Ofayé (see also Gudschinsky, 1971), Koropó, Pataxó, Krenak (Borum), Arikapu, Djeoromitxí and Kamakã (see also Mason, 1950; Ribeiro & van der Voort, 2010; Rodrigues, 2009). Boswood (1973) presented lexical evidence supporting the inclusion of Rikbaktsá in this family. Rodrigues (1986) expanded the internal relations within Macro-Je and ( Rodrigues, 1999) added data from extinct languages.

The inclusion of Bororo in the Macro-Je family, suggested by Guérios (1939), is no longer accepted ( Nikulin, 2020). Rodrigues (1986) included Karirí and Guató in Macro-Je but considered Pataxó a member of the Maxakalí family. Greenberg (1987) classified Guató, Maxakalí, and Pataxó as part of Macro-Je, adding Chiquitano, Otí, and Jabutí. Conversely, Kaufman (1990) excluded Otí, Jabutí, and Karirí from Macro-Je but retained Chiquito (see Nikulin, 2020). For other proposals, see de Castro Alves (2010) and Ribeiro & van der Voort (2010). Ramirez *et al*. (2015) have argued for the similarities found among the languages of the Je, Maxakali, Borum, and Kamakã groups. Nikulin (2020) grouped Maxakalían and Krenakan (perhaps also Kamakanan) in a branch he calls “Transfranciscanan”. He also provided thorough evidence for a larger Macro-Je family, further suggesting more distant relationship with other families such as Chiquitano, which he considers to have a common ancestor with Proto-Macro-Je.

**Figure 1.**
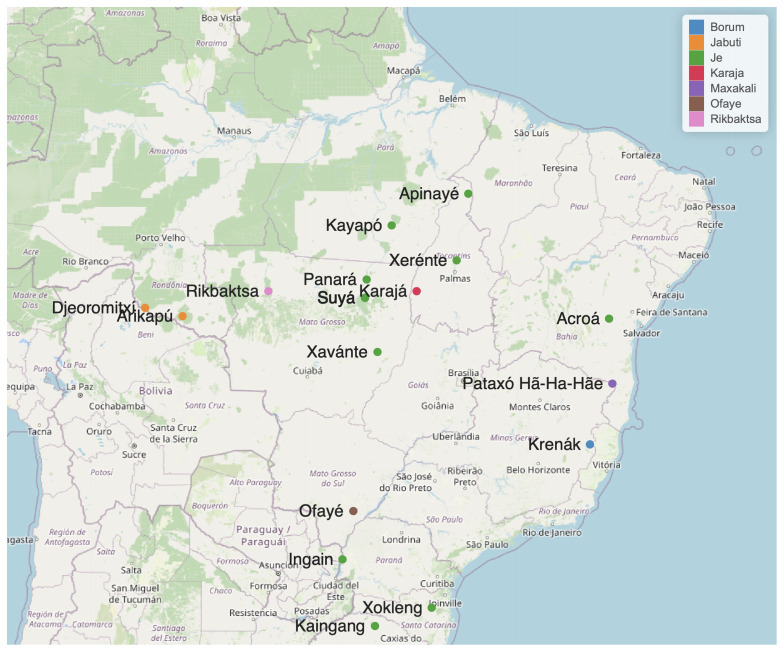
Some Macro-Je languages. The geo-location is based on Hammarström *et al*. (2024).

Table 1 lists 24 languages considered potential members of the Macro-Je family, for which there is some documentation, although limited in some cases. These languages are shown in Figure 1.

**Table 1.** Likely members of Macro-Je.

Doculect	ISO 639	Glottocode	Family
Acroá	acs	acro1239	Central Je
Apinaye	apn	apin1244	Setentrional Je
Arikapu	ark	arik1265	Jabutí
Borum	kqq	kren1239	Borum
Chiquitano	cax	chiq1253	Chiquitano
Djeoromitxí	jbt	djeo1235	Jabutí
Gaviao-Pyhcopji	xri	krik1239	Setentrional Je
Ingain		inga1254	Meridional Je
Kaingang	kgp	kain1272	Meridional Je
Karajá	kpj	kara1500	Karajá
Mebengokre		kara1501	Setentrional Je
Koropó	xxr	coro1248	Maxakalí
Kisedje	suy	suya1243	Setentrional Je
Pataxó	pth	pata1261	Maxakalí
Ofayé	opy	ofay1240	Ofayé
Panara	kre	pana1307	Setentrional Je
Rikbakstá	rkb	rikb1245	Rikbakstá
Suyá	suy	suya1243	Setentrional Je
Tapayuna		beic1238	Setentrional Je
Timbira		timb1253	Setentrional Je
Xacriabá	xkrx	xacr1238	Central Je
Xavante	xav	xava1240	Central Je
Xerente	xer	xer1240	Central Je
Xokleng	xok	xokl1240	Meridional Je

Ribeiro (2012: 266) noted the difficulty in conducting a comparative study of Macro-Je due to the monosyllabic nature of many parallels presented, and the lack of systematic sound correspondences in their roots (see also Ribeiro & van der Voort, 2010). Many of the lexical parallels found among the linguistic families attributed to this stock and referred to as ’cognates’ may be borrowings, given the high probability of interactions that many populations have had since precolonial times with various other populations (see the dates provided in Section 5). The hypothesis that Macro-Je could result from long-term linguistic contact via ethnogenesis ( Dixon & Aikhenvald, 1999) cannot yet be discarded. In this case, similarities found between Je family and some other families / languages, such as Borum and Maxakali, but also Purí and Guató, would be instances of horizontal transfer rather than genetic inheritance ( Ramirez *et al*., 2015).

Evidence from historical-comparative linguistics supports the genetic identity of a ‘nuclear’ Macro-Je linguistic stock, which includes the Je family along with the Chiquitano ( Adelaar, 2008), Jabutí ( Ribeiro & van der Voort, 2010), Ofayé and Karajá ( Ribeiro, 2011; Ribeiro, 2012) families. Conversely, the evidence for including languages such as Bororo, Yatê, Purí, Guató, Karirí, and Otí in the Macro-Je stock is extremely weak.

Speakers of Macro-Je languages inhabit a vast region that extends longitudinally from the Atlantic coast to the Chiquitano Dry Forest and the Guaporé River, and latitudinally from the lower Tocantins to the northern part of the current state of Rio Grande do Sul (see Figure 1). Currently, they number approximately 90,000 individuals, with the most spoken languages being Kaingáng (around 30,000 speakers), Xavante (around 25,000), and Mebengokre (around 13,500).

Typologically, Je languages are characterized phonologically by a rich vowel inventory that includes nasal vowels, which often trigger nasal enhancement phenomena in adjacent segments ( de Carvalho & Damulakis, 2015; Pickering, 2010). The languages have a limited number of possible onsets, typically allowing only sequences of a peripheral obstruent (labial or velar) followed by a rhotic ( Nikulin, 2020). Je languages tend to be head-final SOV as the most common order. Many Je languages display a rare type of split alignment that has not yet been sufficiently studied. This alignment pattern has been called nominative-absolutive ( Gildea & de Castro Alves, 2010; Gildea & de Castro Alves, 2020). Another typical Je feature is the singular-(dual)-plural stem alternation for certain semantic verb types (see Inman & Vuillermet, n.d.), and a somewhat complex system of person marking that interacts with agreement, since most languages are of the dependent marking type. For a more detailed typological profile of the Macro-Je languages, we refer the reader to the works of Salanova (n.d.), Storto (2019: 138–140) (only phonology), Rodrigues (1999) (partially outdated) and Wiesemann (1986).

This paper is structured as follows: Section 2 presents the data used, followed by a presentation of the methods employed in Section 3. The results are discussed in Section 4. A Section on archaeology ( 5) places the Je languages in light of archaeological dates and relates this to the results of the analyses. The paper concludes with final remarks in Section 7.

## 2 Data

The data used in this study is drawn from Ferraz Gerardi (forthcoming), which builds on Nikulin (2020). It consists of 14 languages (given in Table 2) and 516 concepts. The concepts include most Swadesh list items ( Swadesh, 1971) along with many culturally relevant concepts related to fields like agriculture, vegetation and fauna. Given the exploratory nature of this study, cognacy judgments were automatically generated using LexStat ( List, 2014), a state-of-the-art method implemented in the Lingpy Python package ( List & Forkel, 2021). These judgments have not yet undergone manual verification. Nonetheless, Rama & List (2019) demonstrated that automated cognacy judgements can be effectively utilized for preliminary analyses, often yielding reliable results.

**Table 2.** Languages used in this study. Glottocodes, latitude and longitude taken from Hammarström *et al.* (2024).

Doculect	ISO 639	Glottocode	Latitude	Longitude
Apinaye	apn	apin1244	-6.11	-47.63
Arikapu	ark	arik1265	-12.49	-62.73
Canela-Kraho	ram	cane1242	-6.11	-45.13
Gaviao-Pyhcopji	xri	krik1239	-5.94	-46.75
Ingain	-	inga1254	-24.62	-54.25
Kaingang	kgp	kain1272	-27.77	-52.54
Kisedje	suy	suya1243	-11.52	-53.07
Mebengokre	txu	kaya1330	-7.77	-51.67
Panara	kre	pana1307	-10.58	-53
Parkateje	-	timb1254	-	-
Tapayuna	-	beic1238	-	-
Xavante	xav	xava1240	-14.30	-52.44
Xerente	xer	xer1240	-9.59	-48.26
Xokleng	xok	xokl1240	-26.92	-49.59

For the analysis described below, we restricted the dataset to concepts that had corresponding forms in at least 50% of the languages, reducing the set to 303 concepts. Table 3 reveals that approximately 38% of the forms across all languages and remaining concepts are missing. Notably, 210 out of the 622 cognate sets in the dataset are singletons—that is, word forms classified as non-cognate with any other word form. However, 72 of those singletons are found in Arikapu, a Jabutí language and the only non-Je language in the sample ( Ribeiro & van der Voort, 2010). Conversely, five concepts with forms available across all sampled languages are classified as consisting of a single cognate set: ”FOOT”, ”HEAD”, ”LIVER”, ”MEAT” and ”SLEEP”. The full list of concepts and the results of the analyses discussed below are available online ( Ferraz Gerardi *et al.*, 2025).

**Table 3.** Coverage and cognate statistics.

Concepts	303
Languages	14
Words	2638
Coverage	0.622
Cognate sets	622
Singletons	210

## 3 Method

In recent years, the field of computational historical linguistics has experienced substantial growth, with phylogenetic methods adapted from bioinformatics gaining prominence for language classification and dating (see Greenhill (2023) for a comprehensive list of studies). This study seeks to classify the languages in our sample (see Table 2) using Bayesian phylogenetic inference. For the analyses, the data was exported to the NEXUS format ( Maddison *et al*., 1997). In this format, the data is represented as a matrix where each column corresponds to a cognate class, and each row represents a language. The resulting binary representation uses ’1’ to indicate the presence of a cognate class in a language, ’0’ for its absence, and a special character for missing data in a concept-language pair. Following Hoffmann *et al*. (2021), ascertainment correction was applied.

### 3.1 NeighborNet

**Figure 2.**
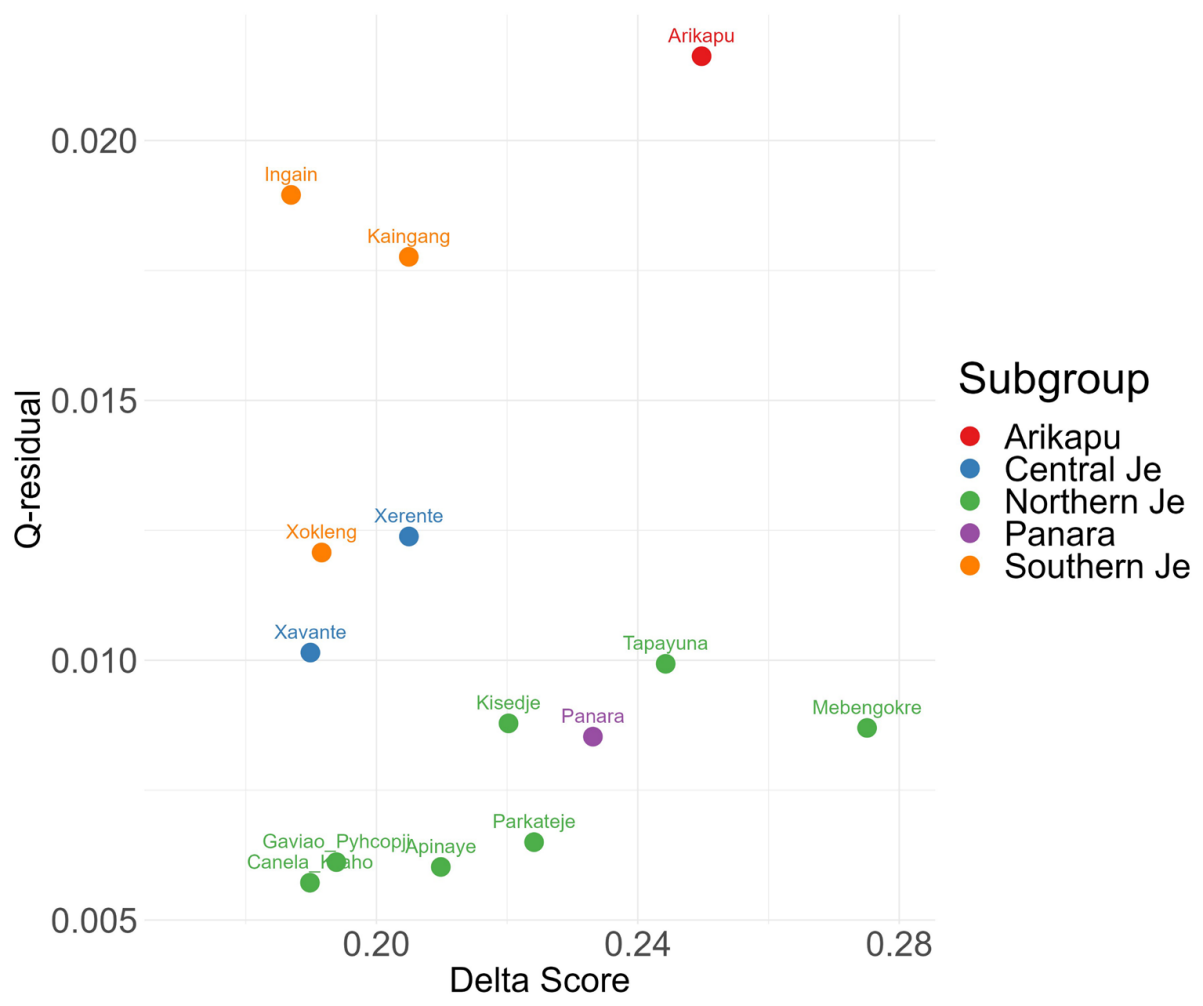
δ-scores and Q-residuals.

**Figure 3.**
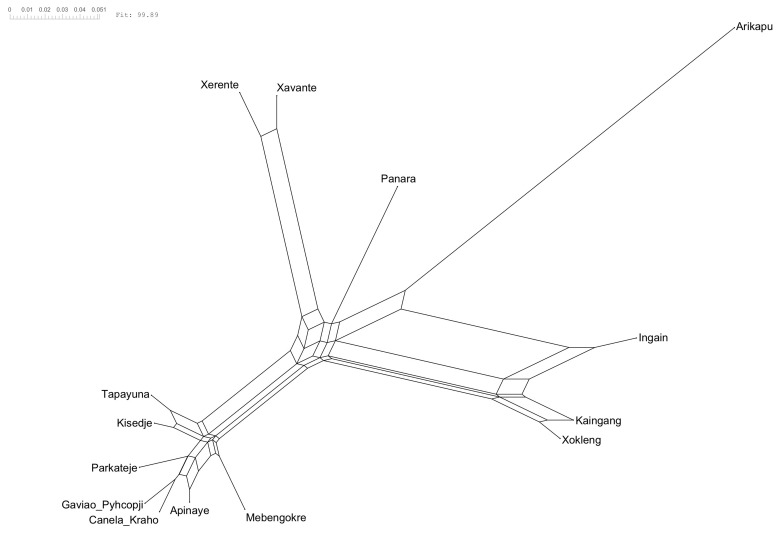
NeighborNet. The reticulations, the ”boxes” between the branches, indicate the conflicting signal in the data.

To quantify and visualize conflicting signals within the dataset, we employed δscores and Q-residuals—indices that measure the tree-likeness of the data ( Bryant & Moulton, 2004; Gray *et al*., 2010). δ-scores range from 0 to 1, with higher values indicating increased conflict, while Q-residuals, adjusted for scaling effects, provide a more direct measure of divergence from a strict tree structure ( Gray *et al*., 2010). Lower Q-residual scores suggest a closer fit to a strict tree. Averaging both scores over the tips yields a measure of overall tree-likeness. These metrics, along with the NeighborNet visualization, were generated using SplitsTree4 ( Huson & Bryant, 2006). Although direct comparison of δ-scores and Q-residuals across different datasets and the evaluation of their significance may not be feasible, low values for these metrics validate the application of methods such as Bayesian phylogenetics, which assume a strict tree structure ( Gray *et al*., 2010). The plot of δ-scores against Q-residuals for each language is given in Figure 2 and the NeighbourNet is given in Figure 3.

The plot in Figure 2 highlights that Northern Je languages (including Panará) and Central Je languages exhibit the least amount of conflicting signal. In contrast, Arikapu—a Jabutí language and the only non-Je language in the sample— exhibits greater conflicting signals and deviates more from a tree-like structure, reflecting its distinct placement within the sample. The Southern Je languages show higher Q-residuals and low δ-scores. This pattern aligns with possible contact with other branches, since the Southern Je groups are the most recent (discussed further below).

### 3.2 Bayesian analyses

Phylogenetic reconstruction was performed using BEAST2 ( Bouckaert *et al*., 2014), testing various binary covarion models ( Huelsenbeck, 2002). Under the binary covarion model, each lexical item, represented as a binary character, can transition between different evolutionary states, such as being subject to change or remaining invariant over time. This flexibility reflects the reality that certain words may evolve at different rates or may be more prone to changes in some languages compared to others, providing a more nuanced and accurate representation of lexical evolution in a phylolinguistic analysis. We tested models differing in whether each concept was assigned an individual substitution rate, allowing for different mutation rates per concept, and whether a strict or relaxed clock was employed. A strict clock assumes a constant rate of evolution across all branches of the tree, while a relaxed clock allows for rate variation, accommodating differing evolutionary dynamics across branches and over time. For all four resulting models, we used a Birth-Death prior ( Gernhard, 2008). Except for a broad root prior, following a uniform distribution from 500 to 6000 years BP, which, based on previous experience, ensured a more stable convergence, we refrained from using calibration points due to limited historical data on the divergence of the languages in our sample. While the separation of Xavante and Xerente approximately 200 years ago ( Maybury-Lewis, 1974) could serve as a calibration point, we excluded it as these languages show minimal variation. According to best practices in tree dating Maurits *et al*. (2020), subfamilies with substantial variation are preferred for calibration.

To identify the best-fitting model, we used Nested Sampling ( Skilling, 2006), which is available as a package for the BEAST2 software ( Russel *et al*., 2018), to calculate the log marginal likelihood for each model. Model selection was performed using the Bayes Factor, which compares the models’ likelihoods and identifies the model that best explains the data ( Hoffmann *et al*., 2021).

We conducted tree inference on the model selected as the best fit based on the Bayes Factor analysis. We performed 10 ^7^ MCMC iterations, sampling a tree every 10 ^3^ iterations, resulting in a posterior sample of 10 ^4^ trees. From these, we obtained a maximum clade credibility tree (MCC) based on common ancestor heights using TreeAnnotator version 2.7.4 ( Heled & Bouckaert, 2013) with a 50% burn-in.

To quantify the quality of the inferred tree topology, we calculated the Generalized Quartet Distance ( Pompei *et al*., 2011) to the Glottolog tree ( Hammarström *et al.*, 2024) using the Quartet package in R ( R Core Team, 2023; Sand *et al*., 2014; Smith, 2019). The Glottolog tree was pruned to include only the languages in our sample. Because Mebengokre, Kaingang, Kisedje and Canela-Kraho are represented as internal nodes rather than tips in the Glottolog tree, we used proxy languages within these nodes to complete the tree for comparison. The Glottocodes of the proxies were kaya1331 (Txucarramae), cent2143 (Central Kaingang), yaru1258 (Yaruma) and krah1246 (Krahô), respectively. Lower GQD values indicate greater topological similarity between the inferred tree and the Glottolog tree.

## 4 Results

The NeighborNet ( Figure 3) and δ-scores and Q-residuals grouped by subgroup ( Figure 2) indicate that most conflicting signals were found for Arikapu and parts of the Southern Je subgroup. However, the mean δ-score of 0.2156 and the mean Q-residuals of 0.01095 indicate generally low levels of conflicting signal across the dataset.

Table 4 summarizes the results of model selection, ranking models by their log-marginal likelihoods. The differences between the highest log-marginal likelihood and those of other models are reported as log Bayes Factors. According to Kass & Raftery (1995), a log Bayes Factor of more than 20 indicates strong support for a model. The results show a clear preference for relaxed clock models over strict clock models. Additionally, models without per-concept substitution models (unpartitioned) and therefore having fewer parameters, are favored. Following model selection, we performed tree inference using the best-fitting model, identified as the unpartitioned relaxed clock model.

**Table 4.** Nested Sampling results (SD = standard deviation of the log-marginal likelihood estimate, BF = Bayes Factor).

Model	Log-Marginal Likelihood	SD	(log) BF
**Unpartitioned**, **relaxed clock**	**-1967.33**	**4.41**	-
Unpartitioned, strict clock	-1991.08	3.21	23.75
Partitioned, relaxed clock	-2048.66	6.62	81.33
Partitioned, strict clock	-2064.12	4.7	96.79

**Figure 4.**
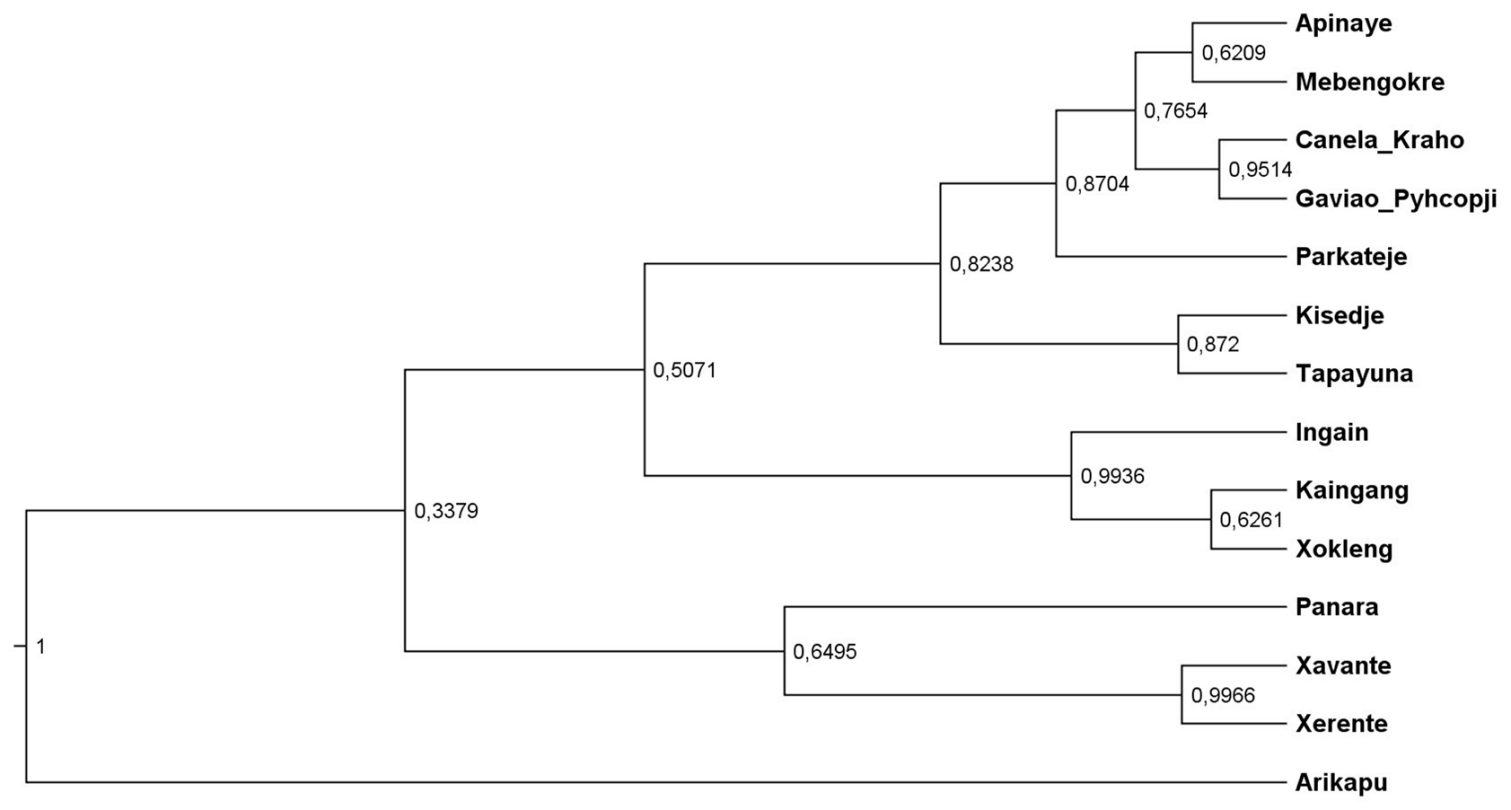
Topology of the tree with unpartitioned concepts and relaxed clock model.

The relative order of splits in the inferred tree ( Figure 4) aligns well with the pre-Columbian history of Je-speaking groups, as reconstructed through archaeological findings.The MCC tree ( Figure 4) of the inferred phylogenetic tree was visualized using the FigTree software ( Rambaut, 2010). The numbers on the nodes correspond to their posterior probabilities. Notably, the inferred tree closely aligns with the established classifications, as quantified by a GQD (Generalized Quartet Distance) to the gold-standard tree of 0.15. In comparison to the findings of Häuser *et al*. (2024), where the GQD was calculated for trees inferred from datasets with expert cognacy judgments, this value is in the low range. Additionally, all alternative models, which had lower likelihoods, produced trees with higher GQD values, further supporting the chosen model.

Interestingly, the lower posterior probabilities in the lower branches of the tree could indicate a dialect continuum situation. It is surprising that the Kaingang-Xokleng clade (similar languages) has a lower posterior value, while the Xavante-Xerente clade (also similar languages) has a high posterior value. A similar pattern is observed between the Apinaye-Mebengokre and Canela-Gaviao clades. The low posterior value in the Panara clade illustrates its uncertainty, as it is usually classified as a Northern Je language.

The first split separates Arikapu (Jabuti) from the Je languages, as expected, followed by a split that separates the Central group. Subsequently, the Northern and Southern languages diverge, with the Southern languages splitting later, consistent with archaeological data (see below).

## 5 Archaeology

The earliest material culture complex commonly associated with Macro-Je speakers is the Una tradition, distinguished by small, simple ceramic vessels found in open-air settlements and rock shelters in central Brazil ( Dias-Jr., 1975; Robrahn-González, 1996). Given its dates and persistence until relatively recently, the Una tradition does not necessarily mark the initial split of the Macro-Je languages, but might have been adopted by various Macro-Je and Je-speaking populations. It does, however, seem to be related to the transition to more sedentary settlements, as well as with the transition to plant cultivation. The earliest dates come from Minas Gerais state, where Una ceramics are associated with early maize and other domesticated plant remains in rock shelters (3490 ± 120 BP, 2 *σ* cal BP 4078-3406 [SI-2788]) ( Bird *et al*., 1991). After ca. 2700 BP, this tradition is found throughout the *cerrado* (central Brazilian savanna) from Goiás to Mato Grosso (2390 ± 60 BP, 2 *σ* cal BP 2699-2147 [Beta-78256], and 2360 ± 70 BP, 2 *σ* cal BP 2698-2133 [SI-4068]) ( Barbosa *et al*., 1982; Wüst, 1998). It is possible that this phenomenon is related to the first split in the tree, separating the Central Je branch ( Figure 4).

The Southern Je branch is clearly associated with the Taquara/Itararé Tradition, based on its spatial distribution, material culture (small, simple ceramic vessels, sometimes with dark burnishing and plastic decoration), burial practices (funerary mounds, sometimes involving cremation), and chronology, all showing a persistence until early colonial times ( Corteletti & Iriarte, 2018; de Mello Araujo, 2007; Noelli & De Souza, 2017). The earliest secure dates for this Tradition reveal a similar horizon of initial settlement for the three southern states, Paraná, Santa Catarina and Rio Grande do Sul - respectively, 1941 ± 35 BP (2 *σ* cal BP 1980-1741 [LACUFF-150057]), 1840 ± 40 (2 *σ* cal BP 1827-1610 [CAMS-53915]) and 1810 ± 85 (2 *σ* cal BP 1890-1431 [SI-813]) ( De Masi, 2001; Parellada, 2016; Schmitz & Brochado, 1972). The dates associated with this archaeological dispersal to the south, later than those of the Una tradition, are coherent with the next split of the tree, which separates the Southern and the Northern Je branches ( Figure 4).

Around the first millennium AD, a new archaeological complex spreads throughout central Brazil and part of the Atlantic coast. Characterized by circular villages with central plazas, urn burials, and large ceramic vessels, this complex is known as the Aratu Tradition ( Robrahn-González, 1996; Wüst & Barreto, 1999). The geographical distribution, similarities with ethnographic ring villages, and chronology confirm that this tradition is related to the Northern and Central Je groups. The origins appear to be in Goiás, with a date of 1220 ± 50 BP (2 *σ* cal BP 1259-960 [Beta-99031]), spreading to the coast of Bahia and Espírito Santo by 1080 ± 90 BP (2 *σ* cal BP 1175-741 [SI-542]) and 1076 ± 36 (2 *σ* cal BP 1050-807 [SI-2347]), respectively ( Calderón, 1969; Perota, 1979; Silva *et al*., 1997). It is important to notice that the presence of this culture in the coast implies that it was not a purely Je phenomenon, but might have been adopted by speakers of other Macro-Je branches.

Similarly, it was probably an innovation shared by both Northern and Central Je speakers, which at this point were already separated ( Figure 4).

## 6 Discussion

The results provide additional support for the existence of geographical clusters within the Je language family, as previously suggested by Rodrigues ( Rodrigues, 1986; Rodrigues, 1999) and Nikulin ( Nikulin & Salanova, 2019: 178). The results also highlight with high certainty that Arikapu is not a Je language.

The inclusion of Panará in a clade with the Central Je languages, specifically Xavante and Xerente, is not entirely unexpected given its geographic location – Panará is usually classified as a separate branch. Historically, this area was in close proximity to the regions where Akwe languages were spoken before their separation around 1815 ( Maybury-Lewis, 1974). In fact, de Carvalho (2016) identified a loanword from an Akwe language in Panará, this being the only known instance of “contact” between these groups. These languages were historically spoken in the southern part of the Brazilian state of Tocantins and in northern Goiás, particularly along the middle course of the Tocantins River.

Nikulin (2020) does not include Panará in the Septentrional branch, instead proposing a separate branch comprising languages spoken in the Goyaz region (see also Nikulin & Salanova, 2019), which form a larger clade together with Panará. Within this branch, Panará is the southernmost language, and Nikulin (2020) acknowledges that part of its lexicon is of unknown origin (see also de Carvalho, 2016). These facts do not contradict the results in the tree presented in Figure 4, on the contrary, together they offer evidence regarding the internal classification.

The closer relationship of the southern clade with the northern clade in the phylogenetic tree is intriguing, especially given that the central group had already diverged while the ancestors of both the northern and southern groups remained united. However, this observation can be substantiated by the archaeological evidence presented in Section 5.

## 7 Conclusion

The phylogeny presented here does not introduce new findings but reinforces previous classifications of Je family, highlighting established geographical clusters of languages as sub-families and raising questions for further inquiry.

In future research, it will be essential to expand the database to attempt a more comprehensive classification that includes a greater number of (non-Je) languages, broader coverage, and manual cognate annotation. Additionally, incorporating not only lexical data but also morphological and syntactic data could be highly valuable, as these elements evolve at different rates and might offer crucial insights into the evolution of a language family ( Hübler & Greenhill, 2022). Another key consideration could be the inclusion of calibration points to construct a dated tree, allowing for comparisons with archaeological timelines.

The geographical clusters (northern, central, and southern groups) provide a realistic model for understanding the spread of Je languages, but they must account for archaeological data, including those for non-Je groups, which are more scarce. While the Macro-Je hypothesis remains valid, further comparative research is required in order to establish a better groundwork for understanding the branching of the phylum. More information about non-Je languages is now available and will be included in future analyses.

## Data Availability

Zenodo: A phylogenetic classification of the Je language family: Supplementary data. https://doi.org/10.5281/zenodo.14615751 ( Ferraz Gerardi *et al*., 2025). The supplementary data is also available on https://github.com/TGH-2020/Je_Phylogeny. This project contains the following underlying data: data/concepts.tsv: Full list of concepts used in this project trees: files to run all model variations with BEAST, as well as the output from the BEAST runs Data are available under the terms of the Creative Commons Attribution 4.0 International license (CC-BY 4.0).
