# Supplementary material for: A phylogenetic classification of the Je language family
Source: Open Res Eur. 2025 May 19;5:29. Originally published 2025 Jan 30. [Version 3] doi: 10.12688/openreseurope.19346.3 (PMC12138499; doi:10.12688/openreseurope.19346.3)
Supplement: Peer review history

# Reviewer report: Reviewer response for version 3

**DOI:** 10.21956/openreseurope.22021.r54231

**Published:** 2025-06-03

**Authors:** Sandra Auderset, Andrés Pablo Salanova

**Affiliations:** 1 Department of Linguistics, University of Bern, Bern, Switzerland; 2 Department of Linguistics, University of Ottawa, Ottawa, Ontario, Canada

This paper presents a fairly standard application of Bayesian phylogenetics to the Jê language family. This family has not been studied with those methods prior and the study is innovative in this regard. There are however some inconsistencies and open questions that should be addressed.

 Section 1:

 The introduction is somewhat confusing and it is not always clear how it relates to the study at hand. Much time is spent discussion various proposals regarding what is to be included in a Macro-Jê phylum, but the study itself focuses on subgrouping within the Jê family.

 The authors bring up several issues in the classification of (Macro-)Jê languages, but it is not always clear whether they think those issues are specific to this family or how their study addressed them. The authors state that "The hypothesis that Macro-Je could result from long-term linguistic contact via ethnogenesis (Dixon & Aikhenvald, 1999) cannot yet be discarded.", suggesting perhaps that this family (or area?) developed differently than others. But the comparison among Macro-Jê languages has proceeded in much the same way as with other language families, and the results don't indicate anything unusual about their history. If anything, scarcity of data and sloppy work are responsible for the slow progress in our understanding of this language family. The problem of disentangling contact from inheritance is ubiquitous in classification and historical linguistics more broadly and not unique to this family. The other issue that caused some confusion on our part concerns the sound correspondences or purported absence thereof.

 The overview of the typology of Jê languages includes a couple of claims that need rephrasing or further explanation.

- "Many Je languages display a rare type of split alignment that has not yet been sufficiently studied. This alignment pattern has been called nominative-absolutive". This is a phenomenon that is exclusive to some Northern Jê languages, that is understood in outline as a pattern that arises from restructuring of a complex construction.
- "a somewhat complex system of person marking that interacts with agreement, since most languages are of the dependent marking type". It is not clear to us what they are referring to here.

As suggested by other reviewers, the Archaeology section (5) should be moved up to the introduction or in any case it needs to appear before the results as it is used for the calibration points.

 Section 2:

 The data that was used as input for the Bayesian phylogenetic analysis is not provided anywhere. It’s stated that it’s extracted from Gerardi & Tresoldi 2024 and a list of the 330 concepts and languages is provided. However, it would helpful to have the actual extracted data set in the supplementary materials.

 The authors should also provide more details regarding the cognate-coding or the checking thereof, as this determines to a large extent how comparable this study is to previous qualitative ones based on the comparative method and sound correspondences. The data in Gerardi & Tresoldi 2024 is already annotated for cognacy. Was that cognate coding carried out automatically? The authors say it was based on Nikulin 2020, which suggests it was done manually based on the comparative method.

 Looking at Gerardi & Tresoldi 2024, it seems that the data was at least partially morpheme-segmented, but the cognate coding is done per concept not per morpheme. Partial cognates are sometimes treated as the same (cf. set 41 for ALLIGATOR), sometimes as different (cf. sets 17 and 15 for ABANDON). All of these methodological issues should be clarified.

 Section 3:

 A burn-in of 50% seems very unusual (it’s usually around 10%). Could the authors add a brief explanation of why it is so high in this case?

 Another question that needs clarification is why the Glottolog tree is used as the ‘gold-standard’ for assessing the quality of the MCC tree of the Bayesian phylogenetic analysis, especially as the tree had to be modified with respect to tips and nodes.

 Section 4:

 Here also it would be good to add some clarifications regarding the interpretation of the results.

- “indicate that most conflicting signals were found for Arikapu and parts of the Southern Je subgroup, whereas Northern Je (including Panará) and Central Je languages exhibit the least amount of conflicting signal.” → But Mebengokre and Tapayuna (Northern Je) exhibit higher Delta scores than the rest of the languages.
- “However, the elevated Q-residuals observed for the Southern Je languages cannot be conclusively attributed to a potential dialect continuum and may instead result from the limited amount of data available for this branch.” →  We don’t fully understand why high Q-residuals need to be interpreted as resulting from a dialect continuum.
- “The relative order of splits in the inferred tree (Figure 4) aligns well with the pre-Columbian history of Je-speaking groups, as reconstructed through archaeological findings.” → This seems a bit circular, as the archaeological findings were used for the calibration points. Also given the difficulties of associating material culture with specific languages, this should phrased more carefully (see also below on Section 5).
- "Excluding individual calibration points resulted in estimates for the omitted timings that remained consistent with those from the full analysis." → Does this mean that only one calibration point is removed at a time? What happens if you remove all calibration points?

The tree in Figure 4 includes several features that go against the consensus on the classification of Jê languages. A summary of what one can claim about the history of Northern Jê based on traditional methodologies can be found in Section 7 of Salanova & Nikulin 2020. We briefly summarize the main points of departure from said consensus:

 (1) Parkatêjê should grouped together with Canela-Krahô and Gavião-Pyhcopji,

 (2) Panará should be closer to the northern languages than to the central ones,

 (3) Khĩsêtjê + Tapayuna should be closer to Mẽbêngôkre + Apinajé.

 A forth issue that might be worth discussing although this is has not reached broad consensus is Nikulin’s (2020) proposal that Khĩsêtjê + Tapayuna are closer to Mẽbêngôkre than Apinajé is, based on shared retentions.

 Section 5:

 The way the archaeological data is interpreted is somewhat wishful: in general terms, the article speaks of splits among branches of the phylum, when archaeological evidence is about expansion. In the case of the Una and the Taquara/Itararé traditions, the expansion is equated with the Macro-Jê and the Southern Jê splits, respectively (the former with reservations). In the case of Aratu, it has to be assumed that there are two branches, split several centuries earlier, that expanded simultaneously with the same material culture.

 General comments:

 In general, the clarity of the paper could be improved with consistent use of spellings of language and family names. Both are variously spelled with and without diacritics – the latter probably out of convenience. The figures would also benefit from more informative and explicit figure captions.

 Some minor issues with terminology/presentation:

- Krenák is in the map, but not the table – or rather it is in the table as Borum, which is also the name of the family(?)
- for Kayapó and Mẽbêngôkre there is an ISO code txu, but it’s not given in the table

- Table 1: would work better if grouped by family
- Figure 1: overlaps of language names and points could be fixed, points could be larger
- Figure 3: color by proposed subgroup like the plot for easier comparison
- Fig 4: shouldn’t this be shared rates tree with relaxed clock, as this was the best model?

If any, are all the source data and materials underlying the results available?

Partly

Is the work original in terms of material and argument?

Yes

Is the work clearly and cogently presented?

Partly

Is the argument persuasive and supported by evidence?

Yes

Does the research article contribute to the cultural, historical, social understanding of the field?

Partly

Does it sufficiently engage with relevant methodologies and secondary literature on the topic?

Yes

Reviewer Expertise:

SA: historical linguistics, phylogenetics; APS: historical linguistics, Jê languages

We confirm that we have read this submission and believe that we have an appropriate level of expertise to confirm that it is of an acceptable scientific standard, however we have significant reservations, as outlined above.

# Reviewer report: Reviewer response for version 3

**DOI:** 10.21956/openreseurope.22021.r54391

**Published:** 2025-05-30

**Authors:** Simon J Greenhill

**Affiliations:** 1 Biological Sciences, University of Auckland, Auckland, New Zealand

I approve the article for indexing.

If any, are all the source data and materials underlying the results available?

Partly

Is the work original in terms of material and argument?

Yes

Is the work clearly and cogently presented?

Yes

Is the argument persuasive and supported by evidence?

Partly

Does the research article contribute to the cultural, historical, social understanding of the field?

Yes

Does it sufficiently engage with relevant methodologies and secondary literature on the topic?

Yes

Reviewer Expertise:

language phylogenies, human prehistory, language evolution

I confirm that I have read this submission and believe that I have an appropriate level of expertise to confirm that it is of an acceptable scientific standard.

# Reviewer report: Reviewer response for version 2

**DOI:** 10.21956/openreseurope.21626.r53106

**Published:** 2025-06-03

**Authors:** Ronald I Kim

**Affiliations:** 1 Adam Mickiewicz University in Poznań, Poznań, Poland

The article under review marks a significant contribution to the rapidly growing field of computational phylogenetic linguistics as well as to the study of the native languages of South America, specifically present-day Brazil. It unquestionably deserves to be indexed and will be read with interest and profit by those working on those fields, as well as historical linguists in general.

 Although I am hardly an expert on computational phylogenetics, the methodology adopted in the study seems both sound and justified, and the authors explain their choices in a mostly clear manner. The results obtained are inherently plausible, although whether they in fact constitute support for phylogenetic groupings is another matter (see below). The discussion of archeological evidence is welcome and appears to support the "real-world" events underlying the diversification of the Je family.

 I am in agreement with the assessment of the previous reviewers, particularly Prof. Salmons, who rightly points out the value of this study for dating the Je family and its subgroups and recommends stressing that point in the conclusions. I also concur with him that diffusion via language contact must always be considered, particularly when dealing with "culturally relevant concepts related to fields like agriculture, vegetation and fauna" which have notoriously spread far and wide across multiple language families, e.g. in prehistoric Eurasia or East Asia. This does not in itself vitiate the authors' analysis, but it would be a good idea to address his question whether the cognacy rates for the Swadesh list and "culturally relevant concepts" are similar, and also to what extent the archeological evidence in section 5 is consistent with "genetic" linguistic subgroups (i.e. a "clean break" in phylogenetic terms), a steadily diversifying dialect continuum, or some combination thereof.

 A more serious issue is Prof. Salmons's comment regarding the comparison of monosyllabic roots. In addition to Ringe's 1992 monograph, see the same author's 1999 article on the relative ease of matching CVC roots. Given that many of the relevant roots in the Je languages are simple CV syllables and that many of the consonant inventories are relatively small, there is a significant risk that automated cognacy judgments will incorrectly identify accidental resemblances as genuine cognate sets, making it all the more difficult to demonstrate greater-than-chance comparisons across these languages. Once again, this does not invalidate the results of the present study, but the authors are urged to address this concern in a footnote, perhaps adding that the cognacy sets obtained by their methodology remain to be (in)validated by the tried and true Comparative Method, which (at least for this "traditional" scholar) remains indispensable to establishing the reality of language families.

 The article is well written, and the English is for the most part idiomatic and stylistically appropriate. A few exceptions are listed below.

 Discussion

 the Macro-Je language phylum's development > the development of the Macro-Je language phylum

 Introduction (p. 4)

 paragraph 3: geographically distributed broadly > with broad geographic distribution

 Meridional > South

 paragraph 4: The proposal for the Macro-Je phylum > The proposal of a Macro-Je phylum

 (Rodrigues, 1999) > Rodrigues (1999)

 paragraph 5: Maxakali > Maxakalí

 paragraph 6: the Macro-Je phylum, for which > the Macro-Je phylum for which

 paragraph 7: many parallels presented, > many claimed cognates,

 p. 5

 Table 1: change Setentrional > North

 change Meridional > South

 Map: why accents on Xavánte, Xerénte?

 Introduction (p. 6)

 paragraph 10: Je languages tend to be head-final SOV as the most common order. > Je languages tend to be head-final and have basic SOV order.

 a somewhat complex system > a complex system

 (only phonology) > (phonology only)

 paragraph 11: no mention of Section 6?

 Results (p. 7)

 paragraph 2: Unsurprisingly Arikapu > Unsurprisingly, Arikapu

 Results (p. 9)

 paragraph 2: Additionally, models without per-concept substitution rates and therefore... > Additionally, models without per-concept substitution rates, and therefore...

 paragraph 3: The MCC tree (Figure 4) of... > The MCC tree of... [since you just referred to Figure 4]

 their posterior probabilities and > their posterior probabilities, and

 Archaeology, paragraph 1: Una tradition > Una Tradition (2x)

 p. 10

 Archaeology, paragraph 2: The earliest secure dates for this Tradition > The earliest secure dates for this tradition

 later than those of the Una tradition, > later than those of the Una Tradition,

 paragraph 3: It is important to notice that the presence of this culture in the coast > It is important to note that the presence of this culture on the coast

 it was probably a retention: how so, and how can a culture be a "retention"? Please explain.

 Discussion, paragraph 1:

 The results also highlight > The results also demonstrate

 paragraph 3: Septentional branch, > Northern branch,

 Conclusion, paragraph 1:

 The phylogeny presented here does not introduce new findings as far as classification is concerned, but reinforces... > The phylogeny presented here reinforces...

 which agrees with archaeological > with agrees with the archaeological

 [In line with the comments above, add another sentence stressing the importance of this agreement for the value of your computational results.]

 Reference cited:

 refer to 1

If any, are all the source data and materials underlying the results available?

Yes

Is the work original in terms of material and argument?

Yes

Is the work clearly and cogently presented?

Yes

Is the argument persuasive and supported by evidence?

Yes

Does the research article contribute to the cultural, historical, social understanding of the field?

Yes

Does it sufficiently engage with relevant methodologies and secondary literature on the topic?

Yes

Reviewer Expertise:

historical linguistics, sociolinguistics, language contact, Indo-European, languages of Eurasia

I confirm that I have read this submission and believe that I have an appropriate level of expertise to confirm that it is of an acceptable scientific standard.

## References

[1] Ringe, Don. 1999. How hard is it to match CVC-roots? Transactions of the Philological Society 97(2): 213–244.

# Reviewer report: Reviewer response for version 2

**DOI:** 10.21956/openreseurope.21626.r53104

**Published:** 2025-05-15

**Authors:** Benedict King

**Affiliations:** 1 Max Planck Institute for Evolutionary Anthropology, Leipzig,, Germany

The study is a fairly standard phylogenetic analysis of a language family. It’s been done well and is clearly written. I have a few points about that analysis, in particular how the calibrations have been implemented. My main reason for selecting approval with reservations is that the position of Panara in the Southern group might be affected by the implementation of the calibrations. Since this is perhaps the most interesting result of the phylogeny, I think it's worth digging into a little more.

  

- In section 3.2 there is the line “testing various covarion models”. It appears that only the standard BEAST implementation of the covarion model is used. What is tested is relaxed vs strict clock and partitioned versus unpartitioned site models.
- The 2 site rate setups used represent extremes: either a single rate for all concepts, or every concept having a different rate (303 extra parameters!). Usually binning by the number of cognate sets (as in the main analysis of Heggarty et al. 2023 for example) provides the best model fit. In an analysis with 303 concepts it seems unlikely that a single rate for all concepts is a good fit.
- The archaeology section should come earlier, to justify the calibration points used in the analysis. This section claims that the archaeological dates are “coherent” with the tree, but isn’t this the evidence used to build the tree?
- I am concerned that the calibrations are not set up in the most logical way, and that there may be an interaction with the tree topology affected Panara. I noticed that in the uncalibrated trees the position of Panara is highly uncertain and switches between North-Central and Southern branches. The justification for the North/Central-South calibration is for an ”archaeological dispersal to the South”, but it’s not clearly described how this should be implemented in a phylogenetic analysis. The calibration is a standard common ancestor node prior at the moment, i.e. it defines the most-recent common ancestor of Je languages. I find it hard to justify a calibration prior without monophyly constraints in language phylogeny. How can you be sure of a link between archaeological culture and language but not be sure that the languages are monophyletic? I find it much more logical to implement calibrations as “originate priors”. This means that the parent node rather than the common ancestor is calibrated. In this case, if there is evidence that the Taquara culture represents the Southern dispersal of the Southern Je languages, then an originate prior would be appropriate to calibrate the time that Southern languages begin independent evolution. This has the advantage that the sister group and therefore the actual node calibrated can be estimated in the phylogeny. I don’t know if Panara should be included as descending from this Southern migration or not, but it sounds like perhaps not.
- From the description in the next paragraph, it sounds like there is no justification for the calibration on North-South group. If this cultural complex is a shared retention, how can it be used to calibrate the common ancestor of the North-Central grouping?
- Briefly summarise Maybury-Lewis for the calibration on Xerente-Xavante. The standard deviation of 0.05 seems way too precise, that’s about 18 days which is shorter than I took to do this review (sorry about that).
- It is not mentioned in the paper if the Bayesian analysis results were checked for proper mixing and convergence and if more than one run was performed. The results do not include the trace log files and only a single tree file.
- It is mentioned that testing for removing calibration points was performed, but no details given and the files aren’t shared
- Another possibly missing detail is that an ascertainment bias correction was used to correct for the absence of all-absent cognate sets.
- In Figure 4 label the 3 groupings. Some indication of which nodes were calibrated might also be good.
- Why is Glottolog considered the gold standard tree for comparison? What is the point of the Bayesian analysis if it isn’t an improvement?
- In the abstract it says the Je family and Macro-Je phylum were investigated. The latter is not really true since only one non-Je language was included, essentially as an outgroup.
- The plain language summary says 516 words were analysed, but only 303 were used in the end.
- In the conclusion it says “Raising questions for further inquiry”. What are these questions?

If any, are all the source data and materials underlying the results available?

Partly

Is the work original in terms of material and argument?

Yes

Is the work clearly and cogently presented?

Yes

Is the argument persuasive and supported by evidence?

Yes

Does the research article contribute to the cultural, historical, social understanding of the field?

Yes

Does it sufficiently engage with relevant methodologies and secondary literature on the topic?

Yes

Reviewer Expertise:

Phylogenetics

I confirm that I have read this submission and believe that I have an appropriate level of expertise to confirm that it is of an acceptable scientific standard, however I have significant reservations, as outlined above.

# Reviewer report: Reviewer response for version 2

**DOI:** 10.21956/openreseurope.21626.r52806

**Published:** 2025-05-14

**Authors:** Natalia Chousou-Polydouri

**Affiliations:** 1 Institute for Mediterranean Studies, Foundation for Research and Technology—Hellas, Rethymno, Greece

The article presents a phylogenetic analysis of Je languages (including one non-Je Macro-Je language) using a large lexical dataset and automatic cognate detection. The article is interesting and provides a much-needed step for the classification of this family. However, several methodological choices need to be clarified and motivated, while other methodological options need to be tested additionally. Finally, some results and discussion points may need to be modified accordingly.

**Methodological Choices**

Coding method

 The coding method needs to be further clarified. If I understand well, these are not cognate classes per se, but partial cognate classes that only include items that belong to the same concept. These have been named with different terms in the literature, such as within-concept cognate sets, or cognate sets without items that have undergone semantic shift, or root-meaning  (or root-concept) sets. Finally each question mark is indicating a missing language-root-concept triplet, not a concept-language pair in the NEXUS format.

Evolutionary model

 It is unclear why only covarion models were tested. Why not a simple binary model or a stochastic Dollo? (even though I can see arguments against a stochastic Dollo, especially in a preliminary analysis). The covarion model was developed for a particular use in evolutionary biology (modeling rate variation due to changes in secondary structure during macroevolution) and it is at best unclear what it would represent in linguistic evolution. The authors' explanation on the appropriateness of the covarion model is inaccurate. First, different words (I assume here roots or concepts are intended) evolving at different rates (a reasonable assumption) should be modeled by rate heterogeneity across sites, not by the covarion model (which is indeed the case in the article). Second, according to the covarion model as used, words that are "prone to change in some languages more than in others", essentially means that the same root appears and disappears relatively rapidly for the same concept for some languages. This is a highly unrealistic situation and does not corresponding to any known linguistic phenomenon.

Tree prior

 The choice of tree prior needs to be motivated and further clarified. Also, additional tree priors need to be tested. There are different parameterizations of BDSKY. Which exactly was used and why? Skyline priors are especially suited for populations dynamics and epidemiology. Why not use a plain birth death tree prior in this study (possibly with incomplete sampling)? All parameters of the model chosen need to be explained (i.e. what they represent in linguistic evolution) and also the specific prior distributions used for each parameter need to be described.

Root prior

 The uniform prior used seems like a rather restrictive prior for the root of the tree, especially since it doesn’t allow any older dates than 6000. Also, the reasoning given for choosing this prior (to improve convergence) is puzzling. Priors are chosen to reflect prior beliefs and they have to be justified. What exactly were the convergence issues and which other prior distributions were tested? Furthermore, in Figure 4, the bar corresponding to the 95% confidence interval for the root age is going all the way to 6000 years before present, further indicating that the root prior is too restrictive.

**Calibrations**

general comments

 The archaeological section needs to be moved to the introductory section of the paper, before the methods and analysis sections. It is not contextualizing the research, it is used as an assumption in order to calibrate the tree. 

 The thought process from the archaeological facts to the derivation of calibrating distributions needs to be expanded.

 Finally, all details regarding the calibrations need to be given in the Methods section. It is not clear exactly how these calibrations were implemented. Did you constrain specific monophyletic groups? Were they crown or stem calibrations? Also, the distribution choice and the values chosen need to be justified. why was the normal distribution chosen here, while the lognormal in the Xerente-Xavante case? if I understand well, in all cases you only have dates that indicate that a split cannot be younger than this date.

Xerente-Xavante split

 The key facts used to derive the calibration from the citation listed need to be repeated here, as well as an explanation of how the calibration was derived (i.e. the thought process).

Northern-Southern and Northern-Center splits

 Here the archaeological facts are presented in detail, but it is unclear how they led to the particular calibrations. Why was the normal distribution chosen here, while the lognormal in the Xerente-Xavante case? if I understand well, in all cases you only have dates that indicate that a split cannot be younger than this date.

**Analysis**

 You should report how many runs were conducted and how many among these converged. A 50% burn-in is a really high percentage and indicates that you probably have not reached convergence. In any case longer runs are needed for reliable results.

**Results and Discussion**

general comment

 Throughout the results and discussion section it needs to be clear what is assumed and what is a result of your analysis. For example, were there any subgroups that were assumed to be monophyletic during the calibration process?

 Another case is the end of the archaeology section (section 5). It seems here that you are interpreting the archaeology based on your tree, while you have used the archaeological data to calibrate it.  It needs to be clear what is assumed in order to derive these calibrations, both in terms of which cultural tradition is associated with which branch, as well as which branches (if any) are assumed to be monophyletic in the analysis.

 In the conclusion (section 7), the reasoning seems circular. The archaeological data were used to calibrate the phylogeny, so you can’t interpret the dating as fitting with the archaeology. If the dating was done with linguistic data (e.g. through old sources), then a comparison with archaeological dates would be appropriate.

non-tree-like signal of Arikapu

 This is not a self-evident result, as indicated at the end of page 7. If anything, the default expectation would be that Arikapu would be widely separated from everything else, since it is a more distant relative. It would not have necessarily more conflicting signal. Instead, I think this may be because of unsure/erroneous cognate judgements between Arikapu and the rest of the languages, exactly because of the distant relationship. Automated cognate judgements for Arikapu need to be evaluated separately to see if they are significantly different from manual cognate judgements and/or all Arikapu judgments should be reviewed by hand.

Bayes Factors comparisons

 The text here (end of page 7 and beginning of page 9) is contradictory and also does not agree with Table 4. On the table the model with shared rates is highlighted (presumably as the chosen one), but in the text you mention using per-concept rates. Please clarify which model was used for the final analysis.

 Additionally, the way that BF factors are reported in table 4 is confusing. Positive logBF should be associated with the chosen model, not with the non-chosen ones. A 4X4 table with all comparisons would be better.

**MCMC tree**

 The calibrated splits should be annotated on the tree, as well as any clades that are used in the results and discussion (e.. Norther, Central etc).

**Minor corrections**

 - Rikbaktsa is mispelled two times in Table1

 - page 6, first column, second paragraph. The sentence starting "Je languages tend to be head-final" is ungrammatical. Maybe there is a "with" missing between head-final and SOV?

 - Inman & Vuillermet is published, see www.degruyterbrill.com/document/doi/10.1515/lingty-2023-0040/html?lang=en

 - page 10, first column, last paragraph. It is unclear what Akwe languages refers to. Is it a cover term for Xavante and Xerente? Without more context, this section’s argument is not entirely clear.

If any, are all the source data and materials underlying the results available?

Yes

Is the work original in terms of material and argument?

Yes

Is the work clearly and cogently presented?

Partly

Is the argument persuasive and supported by evidence?

Partly

Does the research article contribute to the cultural, historical, social understanding of the field?

Yes

Does it sufficiently engage with relevant methodologies and secondary literature on the topic?

Partly

Reviewer Expertise:

phylogenetics, historical linguistics

I confirm that I have read this submission and believe that I have an appropriate level of expertise to confirm that it is of an acceptable scientific standard, however I have significant reservations, as outlined above.

# Reviewer report: Reviewer response for version 2

**DOI:** 10.21956/openreseurope.21626.r52803

**Published:** 2025-05-03

**Authors:** Robin J. Ryder

**Affiliations:** 1 Université Paris Dauphine and Crest INSEE, Paris, France

The authors reconstruct the history of the Je languages, using phylolinguistics methods on lexical data.

 This paper is an interesting and relevant addition to the literature and is clearly written. The revised version follows the state of the art; the statistical methods and conclusions seem robust. The authors used automatic cognate detection to transform the lexical data into binary data: their results tend to show that this procedure is appropriate for this data set. The online supplementary material includes the necessary XML files to reproduce the studies, as well as the trees sampled from the posterior distribution.

 I recommend accepting this paper. I only have a small number of minor comments:

 * You could check that the results are stable when you restrict the data to only concepts in the Swadesh list. The rationale is that those concepts are assumed to change at a slower rate and to be less susceptible to borrowing, thus giving more reliable signal on the deep history.

 * Although we are given details of the MCMC, no diagonistics are given. Please specify how you checked the MCMC has converged and mixed well (i.e. that the number of iterations is large enough).

 * The calibration of the Xerente-Xavante split has mean 200 and standard deviation 0.05. That is a very low sd; is it maybe a typo?

 * The subfamilies are sometimes referred to as Northern and Southern, sometimes as Septentrional and Meridional. Please use consistent names. Also, there is a missing p in Septentrional in Table 1.

 * I was a bit confused by the different lists of languages. Table 1 is the most complete list, yet it doesn't include Canela-Kraho and Parkateje (both used in the phylogenies) nor Kayapo and Krenak (both present on the map of Figure 1). Additionally, the code for Mebengokre is different between Table 1 and Table 2.

 * I was very glad to read the last paragraph of section 6 on reconstructing calibration: this check strengthens your conclusions. But the paragraph is vague. You should include the details of the reconstructed ages (for example with a figure such as those in Pellard, Ryder & Jacques 2024 or in Ryder & Nicholls 2011, two papers in which we argue for that practice).

 Refer:Pellard, T., Ryder, R., & Jacques, G. (2024). The family tree model. The Wiley Blackwell companion to diachronic linguistics.

If any, are all the source data and materials underlying the results available?

Yes

Is the work original in terms of material and argument?

Yes

Is the work clearly and cogently presented?

Yes

Is the argument persuasive and supported by evidence?

Yes

Does the research article contribute to the cultural, historical, social understanding of the field?

Yes

Does it sufficiently engage with relevant methodologies and secondary literature on the topic?

Yes

Reviewer Expertise:

phylolinguistics, stochastic modelling, Bayesian inference, computational statistics

I confirm that I have read this submission and believe that I have an appropriate level of expertise to confirm that it is of an acceptable scientific standard.

## References

[1] : Missing Data in a Stochastic Dollo Model for Binary Trait Data, and its Application to the Dating of Proto-Indo-European. *Journal of the Royal Statistical Society Series C: Applied Statistics* .2011;60(1) : 10.1111/j.1467-9876.2010.00743.x 71-92 10.1111/j.1467-9876.2010.00743.x

# Response

**Published:** 2025-05-14

**Authors:** Tim Wientzek-Paul

**Affiliations:** University of Tubingen Department of Linguistics, Tübingen, Baden-Württemberg, Germany

We thank the reviewer for the helpful and constructive comments. Since the paper has been accepted, we have limited the revisions to minor corrections and clarifications. We have checked MCMC convergence using Tracer 1.7.2 (Rambaut et al., 2018), and most parameters show effective sample sizes above 200, in line with the rule of thumb in Hoffmann et al. (2021). Regarding the calibration prior, the mean of 200 refers to real space, while the standard deviation of 0.05 is specified in log space, as indicated in the manuscript. We have revised the terminology for the subfamilies to consistently use “Northern” and “Southern” throughout. We also added the missing languages to Table 1 and clarified that “Mebengokre” and “Kayapó” refer to the same variety. While we hope that our manual concept selection and cognate coding helped minimize the impact of borrowing, we agree that restricting analyses to Swadesh concepts could be a valuable robustness check, and we will consider this in future work. Similarly, we appreciate the suggestion to report reconstructed calibration ages in more detail and will take this into account in upcoming projects. **References:** Rambaut, A., Drummond, A.J., Xie, D., Baele, G. & Suchard, M.A. (2018). Posterior summarisation in Bayesian phylogenetics using Tracer 1.7. *Systematic Biology*. **syy032**. doi:10.1093/sysbio/syy032 Hoffmann, K., Bouckaert, R., Greenhill, S. J., & Kühnert, D. (2021). Bayesian phylogenetic analysis of linguistic data using BEAST. *Journal of Language Evolution*, *6*(2), 119–135. https://doi.org/10.1093/jole/lzab005

# Reviewer report: Reviewer response for version 2

**DOI:** 10.21956/openreseurope.21626.r52808

**Published:** 2025-04-19

**Authors:** Joseph Salmons

**Affiliations:** 1 University of Wisconsin, Madison, USA

This article provides a welcome contribution to the classification of the Je languages and will be of interest to comparative linguists generally. It deserves to be out there, and I simply have some suggestions for clarifying and strengthening it.

 The authors rightly stress the value of this paper for dating developments in the family, and dating is often a particularly challenging issue — like the large literature on this for Indo-European shows. That said, it’s also important that we have independent converging evidence for this picture of the family. First, the phylogenetic evidence is consistent with traditional comparative work. Second, it appears consistent with the archeological record. These three perspectives together give us greater confidence in the overall picture. You might stress that positive rather than concluding with the negative that you don’t present ‘new findings’.

 My main concern is an issue noted in passing in the manuscript and underscored by the first reviewer, that many of the forms used are monosyllabic so that accidental correspondences come into play. In fact, a lot of the forms include only a single consonant, a CV syllable. Ribeiro (2012), cited on this, is more explicit about this than the paper is. The reader should have more details about this issue and how important it is. To go beyond Ribeiro: At least some of these languages have small phoneme inventories, like 13 or 14 consonants, where the modal number of consonants reported across languages is 21 (Gordon 2016: 45, drawing on Maddieson 1984). This means there’s a high likelihood of accidental correspondences, even assuming perfectly systematic sound correspondences and leaving aside borrowings. This presumably risks overstating the relatedness of these languages. Campbell (1988) and Ringe (1992) provide two different discussions of this issue for comparative linguistics.

 A second issue is language contact. Yes, borrowing surely plays a role here, especially as the subgrouping lines up with geography, so that many sisters would have often been in contact with each other longer than with more distant relatives. ‘Culturally relevant concepts’ are included in the dataset here, and those are often seen as prone to borrowing, while the traditional claim was that Swadesh items were less likely to be borrowed. (The Leipzig-Jakarta list and other more recent lists have better empirical foundations, but Kossmann 2013 shows how complex the issue is.) Are the cognacy rates similar for the Swadesh and cultural sets?  This is an important area for future work, but some initial evidence would be helpful here.

 Finally, you talk about using 516 items, but 303 is the actual number of comparisons used. I would change that for clarity while noting that you started from a larger set.

 Refer:

1. Campbell, Lyle. 1988. Review article on Greenberg 1987. Language. 64(3). 591-615.
2. Gordon, Matthew K. 2016. Phonological typology. Oxford: Oxford University Press.
3. Kossmann, Maarten. 2013. The Arabic influence on northern Berber. Leiden: Brill.
4. Maddieson, Ian. 1984. Patterns of Sounds. Cambridge: Cambridge University Press.
5. Ringe, Donald A. 1992. On calculating the factor of chance in language comparison. Philadelphia: Transactions of the American Philosophical Society 82(1).

If any, are all the source data and materials underlying the results available?

Yes

Is the work original in terms of material and argument?

Yes

Is the work clearly and cogently presented?

Yes

Is the argument persuasive and supported by evidence?

Yes

Does the research article contribute to the cultural, historical, social understanding of the field?

Yes

Does it sufficiently engage with relevant methodologies and secondary literature on the topic?

Yes

Reviewer Expertise:

historical and comparative linguistics

I confirm that I have read this submission and believe that I have an appropriate level of expertise to confirm that it is of an acceptable scientific standard, however I have significant reservations, as outlined above.

# Response

**Published:** 2025-05-14

**Authors:** Tim Wientzek-Paul

**Affiliations:** University of Tubingen Department of Linguistics, Tübingen, Baden-Württemberg, Germany

*We are grateful to the reviewer for taking the time to read our piece and contributing with observations and suggestions.* *The main concern voiced by the reviewer targets the short (CV and CVC) shape of most of the compared lexical elements.*

*He brings up the well-known fact that putative cognate sets of this kind can be problematic when advancing claims of common ancestry among languages, as a prevalence of short morpheme shapes leaves one unable to exclude chance as the main factor explaining the attested similarities. Further, the reviewer notes that similar concerns were expressed in Ribeiro (2012), a work cited in our paper. Despite the cogent nature of these observations, they are, as far as an evaluation of the present paper goes, entirely beside the point and misplaced, as we explain now.*

*Although the hypothesis of a Macro-Jê family, a group of languages and families related to the Jê linguistic family, remains essentially open as far as its ‘definitive’ nature and limits are concerned (see e.g., Rodrigues 1999; Ribeiro & van der Voort 2010; Nikulin 2020), the same is not true for the Jê language family, which constitutes the focus of the paper. Indeed, Davis (1966), even having at his disposal much less extensive data than is currently available (his comparative Jê is limited to 112 cognate sets, among lexical and grammatical morphemes), describes the languages in question as ‘‘obviously related’’ (Davis 1966: 10). See also Ribeiro & van der Voort 2010: 549).*

*Unsurprisingly, the discussion in Ribeiro (2012), mentioned by the reviewer, concerns the status of Macro-Jê, not Jê, as a family (see Ribeiro 2012: 266). The existence of a Jê language family has been undisputed since the middle of the 20th century.* *The only non-Jê, Macro-Jê language in the study is Arikapu, a member of the Jabuti family used as an outgroup. The language, along with its closer relative Djeromitxi, is also clearly related to the Jê family, as shown by shared morphological aberrancies involving cognate sets supported by regular correspondences (Ribeiro & van der Voort 2010: 533); paradigmatically structured morphology (Ribeiro & van der Voort 2010:563-4) and shared (inherited) homophonies (e.g., Proto-Jê ŋɾɛ ‘dance’, ŋɾɛ ‘egg’ : Arikapu ɾɛ̃ ‘dance’, ɾɛ̃ ‘egg’; Ribeiro & van der Voort 2010: 562-3).* *That said, we agree with the general gist of the reviewer’s focus on the nature of cognate judgements underlying our analysis. An absolutely necessary follow-up to the present paper will implement a manual checking of the automatic cognation judgements on which our contribution was based. The same applies, of course, to the other source of similarities: borrowing/diffusion.*    

**References:** Davis, Irvine. 1966. Comparative Jê phonology. *Estudos Lingüísticos: Revista    Brasileira de Lingüística Teórica e Aplicada* 1 (2): 10-24. Ribeiro, Eduardo R. 2012. A Grammar of Karajá. Doctoral Dissertation, The         University of Chicago. Ribeiro, Eduardo R. and Hein van der Voort. 2010. Nimuendajú was right: the        inclusion of the Jabutí language family in the Macro-Jê stock. *International    Journal of American Linguistics* 76 (4): 517-570. Rodrigues, Aryon D. 1999. Macro-Jê. In: R. M. W. Dixon and Alexandra Aikhenvald           (eds.) *Amazonian Languages*. Cambridge: CUP: 165-206.

# Reviewer report: Reviewer response for version 2

**DOI:** 10.21956/openreseurope.21626.r52440

**Published:** 2025-04-12

**Authors:** Simon J Greenhill

**Affiliations:** 1 Biological Sciences, University of Auckland, Auckland, New Zealand

I think the authors for their considered response to my comments and suggestions. I thnk this paper is now suitable for indexing

If any, are all the source data and materials underlying the results available?

Partly

Is the work original in terms of material and argument?

Yes

Is the work clearly and cogently presented?

Yes

Is the argument persuasive and supported by evidence?

Partly

Does the research article contribute to the cultural, historical, social understanding of the field?

Yes

Does it sufficiently engage with relevant methodologies and secondary literature on the topic?

Yes

Reviewer Expertise:

language phylogenies, human prehistory, language evolution

I confirm that I have read this submission and believe that I have an appropriate level of expertise to confirm that it is of an acceptable scientific standard.

# Reviewer report: Reviewer response for version 1

**DOI:** 10.21956/openreseurope.20937.r50634

**Published:** 2025-02-06

**Authors:** Simon J Greenhill

**Affiliations:** 1 Biological Sciences, University of Auckland, Auckland, New Zealand

This paper presents a phylogenetic analysis of the Je languages. The language family is interesting, and the analysis looks to follow best practices. Overall the paper is well-constructed and I only have few suggestions to improve the paper (of which I consider #1 and #2 the most critical).

 I recommend acceptance after these revisions.

 1. No evaluation of cognate accuracy.

 The authors apply an automated cognate detection algorithm to identify cognates, making the argument that these automated methods perform well.

 However, automated cognates are not perfect, and not as widely accepted as the current manuscript suggests. This is especially the case when -- as the paper notes -- there has been (a) reported problems identifying cognates due to monosyllabic words, and (b) high levels of borrowing.

 How can the authors show us that the cognates they are basing their analyses off are remotely plausible? I would like to see a more careful evaluation on a subset of the data: are there some cognates identified in the literature using traditional methods? do the automated ones recapitulate these? even better, .is there a cognate-coded subset that can be used as a formal test of how well the automated approach is at capturing true cognates? Failing this, can the authors present some of the identified sets and convince the reader that they are plausible?

 Adding the identified cognate sets into the supplementary material would also help readers evaluate their performance. (I commend the authors on having everything else in the supplement).

 2. Why not date the language family?

 Despite using a method that estimates time-dated phylogenies, the analysis does not add any time information. The paper would be much more impactful if it added date calibrations into the analysis. The authors state that they can't add time as there is only one calibration -- Xavante & Xerente ~200B.P. -- but this does not meet the criteria from Maurits et al.

 I disagree. First, the Maurits et al. article is overstating the case quite substantially, and they are basing their recommendations on a very well-documented and well-studied language group (Uralic): quite a different situation to Macro-Je. Second, the section on archaeology goes on to describe a range of other plausible dates that look like ready calibrations to me e.g. Southern Je, ~1800-2000 B.P. and the Aratu tradition ~1200B.P.

 3. The authors suggest that the low delta/q-residual scores in the southern Je clade indicate a possible dialect chain. An alternative is that there is something different about these data that make the scores higher. This could be higher levels of missing data, or could be fewer identified cognate sets due to some linguistic process. Can these alternatives be ruled out?

 4. The paper opens with “Comprises about 15 languages”…but table 1 lists far more which threw me on first reading. To avoid reader confusion, I recommend introducing Macro-Je in the introduction before focusing in on the Je subfamily. Consistency with terms would help as well (e.g. Macro-Je = family, Je = subfamily).

 5. The paper opens by talking about phylogenetic methods. This makes the paper seem like it'll be a technical evaluation of these tools, but this is not the case. It also seems like the only reason for doing this analysis is to apply phylogenetic methods rather than shedding light on human prehistory. Can the opening paragraph reframe around the question of Je/Macro-Je rather than the methods? The paper will get more readers this way.

 6. The term 'partitioned' is used in the tables, but this is not explained in text. I would recommend a term like "per-word rates" or defining this when you describe the models.

If any, are all the source data and materials underlying the results available?

Partly

Is the work original in terms of material and argument?

Yes

Is the work clearly and cogently presented?

Yes

Is the argument persuasive and supported by evidence?

Partly

Does the research article contribute to the cultural, historical, social understanding of the field?

Yes

Does it sufficiently engage with relevant methodologies and secondary literature on the topic?

Yes

Reviewer Expertise:

language phylogenies, human prehistory, language evolution

I confirm that I have read this submission and believe that I have an appropriate level of expertise to confirm that it is of an acceptable scientific standard, however I have significant reservations, as outlined above.

# Response

**Published:** 2025-03-18

**Authors:** Tim Wientzek-Paul

**Affiliations:** University of Tubingen Department of Linguistics, Tübingen, Baden-Württemberg, Germany

Dear Simon, we appreciate your valuable feedback which we consider to have improved the paper. We have followed your recommendations, and the article has been revised accordingly.

**1) No evaluation of cognate accuracy.**

 This is an excellent suggestion. To assess the reliability of the automated cognate detection, we manually annotated a subset of the data and calculated a B-Cubed score. The results indicate that the automatic method produces highly plausible cognacy judgments. Additionally, we have included the identified cognate sets in the Supplementary Material to allow readers to evaluate them directly.

**2) Why not date the language family?**

 Following your recommendation, we have repeated the Bayesian analysis using calibration points. This resulted in a slight reordering of splits, with Southern Je now diverging from Proto-Central-Northern Je instead of Central Je splitting first (a split with a very low posterior value in the un-dated tree). The updated results are reflected in the revised Figure 4 and Table 4.

**3) Low delta/q-residual scores in the Southern Je clade.**

 We investigated alternative explanations for the higher Q-residuals observed for the Southern Je clade and found that there were indeed higher levels of missing data than for all other clades. We adjusted our interpretation of the results accordingly.

**4) Macro-Je and Je confusion**

 We agree that the previous terminology was inconsistent and potentially confusing. To improve clarity, we now refer to Macro-Je as a “phylum” and Je as a “family” throughout the manuscript.

**5) The paper opens by talking about phylogenetic methods.**

 We have chosen not to address this point.

**6) The term 'partitioned' is used in the tables, but this is not explained in text.**

 Thank you for pointing this out. We have replaced the terms "partitioned" and "unpartitioned" with the more intuitive labels "per-concept" and "shared" when describing substitution rates.

# Response

**Published:** 2025-03-28

**Authors:** Simon Greenhill

**Affiliations:** Biological Sciences, The University of Auckland, Auckland, Auckland, New Zealand

The authors have handled my comments and suggestions appropriately and I have no further concerns.
